# Characterisation and prevalence of inherited retinal diseases in the Finnish population reveals enrichment of population-specific phenotypes and causative variants

**DOI:** 10.1136/bjo-2025-327427

**Published:** 2025-06-26

**Authors:** Laura Lähteenoja, Pasi Ohtonen, Aura Falck, Elisa Johanna Rahikkala

**Affiliations:** 1 Research Unit of Clinical Medicine and Medical Research Center Oulu, Oulu University Hospital and University of Oulu, Oulu, Finland; 2 Clinical Genetics Unit and Ophthalmology Unit, Oulu University Hospital, Oulu, Finland; 3 Research Service Unit, Oulu University Hospital, Oulu, Finland; 4 Ophthalmology Unit, Oulu University Hospital, Oulu, Finland; 5 Clinical Genetics Unit, Research Unit of Clinical Medicine and Medical Research Center Oulu, Oulu University Hospital and University of Oulu, Oulu, Finland; 6 Genomics Unit, Laboratory Division, Tyks Turku University Hospital, Turku, Finland

**Keywords:** Retina, Dystrophy, Genetics, Epidemiology, Diagnostic tests/Investigation

## Abstract

**Aims:**

This study aims to assess clinical and genetic characteristics as well as the prevalence of inherited retinal dystrophies (IRD) and their subphenotypes in the Finnish founder population.

**Methods:**

A retrospective analysis of clinical and genetic data from Northern Finnish patients diagnosed with IRD between 1996 and 2023 at Oulu University Hospital, Finland, was conducted.

**Results:**

The cohort comprised 582 patients with IRD, categorised into 16 different subphenotypes. Pathogenic or likely pathogenic variants explaining IRD were identified in 36% (n=210/582) of all patients and 80% (n=210/261) of genetically tested patients with IRD. Diagnostic yields varied between different IRD subphenotypes. The genetic aetiology was most commonly confirmed in X-linked retinoschisis, severe early childhood-onset retinal dystrophy, congenital stationary night blindness and choroideremia. The lowest rates of causative variant identification were observed in cone or cone-rod dystrophy and macular dystrophy. In total, 70 pathogenic or likely pathogenic variants were identified across 39 different genes; variants in the *FZD4* and *RPGR* genes were the most prevalent. Over half of the variants were enriched in the Finnish population. The estimated total prevalence of IRDs in Northern Finland was 69.8/100 000 (1:1432). The prevalence of the most common subphenotypes was as follows: retinitis pigmentosa, 25.3/100 000; X-linked retinoschisis, 10.7/100 000; Usher syndrome, 8.9/100 000; choroideremia, 7/100 000 and cone or cone-rod dystrophy, 6/100 000.

**Conclusion:**

The Northern Finnish population exhibits an enrichment of population-specific IRD-associated variants, resulting in a high overall prevalence of IRDs and an increased prevalence of selected retinal subphenotypes, such as retinoschisis, choroideremia and Usher syndrome types 3 and 1.

**WHAT IS ALREADY KNOWN ON THIS TOPIC**

- Inherited retinal dystrophies (IRDs) are highly heterogeneous and demonstrate population-specific distributions of pathogenic variants and subphenotypes.

**WHAT THIS STUDY ADDS**

- Our study identified an enrichment of population-specific IRD-associated variants, resulting in high prevalence rates of subphenotypes, including retinoschisis, choroideremia and syndromic IRDs such as Usher disease types 1 and 3, as well as gyrate atrophy. Diagnostic yields of genetic testing varied significantly between different subphenotypes.

**HOW THIS STUDY MIGHT AFFECT RESEARCH, PRACTICE OR POLICY**

- The Finnish population bears a high burden of genetic variants leading to IRDs, resulting in an increased prevalence of certain retinal subphenotypes.

## Introduction

 Inherited retinal dystrophies (IRDs) are a heterogeneous group of conditions characterised by progressive or stationary retinal dysfunction due to pathogenic variants encoding proteins critical for normal retinal function. IRDs are a leading cause of vision loss among the working-age population.^1^ These visually disabling conditions impose considerable psychological and economic burdens on affected individuals and their families. People with IRDs often experience early-onset vision loss, leading to a decline in quality of life.^2 3^ Depending on the population studied, IRDs affect approximately 1 in 2000–4000 individuals.^1^

IRDs can be classified as either syndromic or non-syndromic, and over 300 different causative genes have been identified (Retnet, https://retnet.org/). Phenotypic heterogeneity adds further complexity, as individuals carrying pathogenic variants in the same gene may exhibit different retinal subphenotypes.^1^ A recent German study using targeted next-generation sequencing (NGS) reported molecular diagnostic rates for IRDs ranging from 35% to 95%, depending on the clinical entity. High detection rates were observed for achromatopsia, retinoschisis and choroideremia, while central areolar choroidal dystrophy and macular dystrophy (MD) had the lowest detection rates.^4^ A recent systematic review and meta-analysis of 105 publications found that the current diagnostic yield of NGS for IRDs ranges between 52% and 74%.^1^

This study aimed to investigate the clinical and genetic characteristics, as well as the prevalence, of IRD in the Finnish population. To achieve this, we compiled a comprehensive, population-based registry of 582 Finnish patients diagnosed with IRD.

## Materials and methods

### Subjects

The study population consisted of patients diagnosed with IRDs at Oulu University Hospital, Finland, over a 27-year period from 1996 to 2023. The tertiary catchment area of Oulu University Hospital includes all four central hospitals in Northern Finland and geographically covers 51% of the country. Diagnoses were identified using the International Statistical Classification of Diseases (ICD-10) codes for hereditary retinal dystrophy (H35.5), X-linked retinoschisis (XLRS) (Q14.10), hereditary choroidal dystrophy (H31.2), gyrate atrophy (E72.4), neuronal ceroid lipofuscinoses (NCL) (E75.4), Cockayne syndrome (Q87.11) and Bardet–Biedl syndrome (Q87.81).

Clinical data and genetic laboratory results from routine clinical testing were retrospectively collected from patient records. IRD phenotypes were determined based on the clinical data, and for patients who underwent genetic testing, details of the test used were recorded.

**Figure 1:**
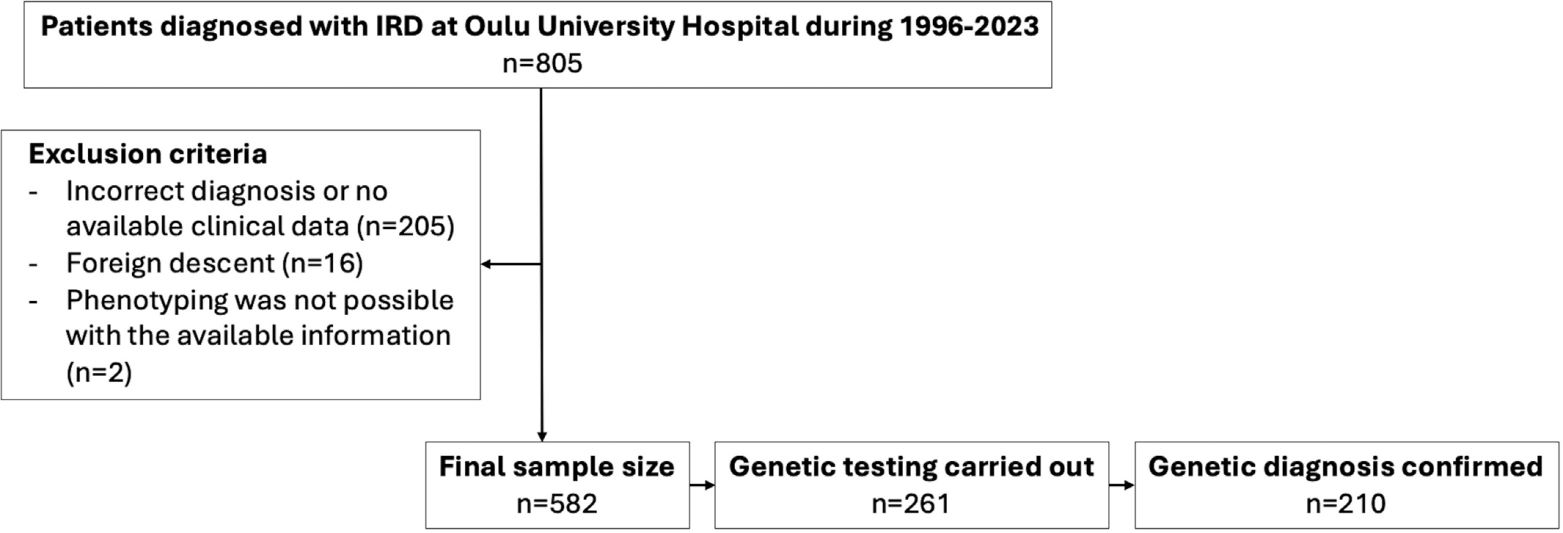
Flowchart of the study population. IRD, inherited retinal dystrophy.

Initially, 805 patients were identified as having an ICD-10 diagnosis code associated with IRDs between 1996 and 2023. Of these patients, 205 were excluded due to erroneous ICD-10 coding or a lack of confirmatory clinical data available in a retrospective review of electronic patient records (figure 1). 16 additional patients were excluded due to foreign descent, and two were excluded because phenotyping was not possible with the available information. Among the excluded Finnish patients, 49% (n=101/207) were deceased. The final study population comprised 582 patients, of whom 45% (n=261/582) had undergone genetic testing for IRDs.

### Clinical evaluation

For simplicity, the phenotypes of all 582 patients were categorised under the supervision of an IRD specialist (AF, 25 years of experience in ophthalmology), based on a retrospective analysis of patient records, into 16 types: retinitis pigmentosa (RP), Leber’s congenital amaurosis (LCA), severe early childhood-onset retinal dystrophy (SECORD), cone dystrophy (CD) or cone-rod dystrophy (CRD), congenital stationary night blindness (CSNB), choroideremia (CHM), achromatopsia, MD, Stargardt disease, Best disease, XLRS, familial exudative vitreoretinopathy (FEVR), Wagner syndrome, Usher syndrome and other syndromic IRDs.

### Genetic testing and data analysis

Genetical testing was conducted on 45% (n=261/582) individuals. The most commonly used genetic test for identifying the genetic aetiology of IRD was the retinal dystrophy NGS gene panel, applied in 41% (n=108/261) of the genotyped cases. The gene panels performed varied depending on the laboratory where the test was conducted and the year the test was ordered. The number of genes included in the NGS panels varied between 105 and 351 genes. When a causative gene variant had been previously identified in a family, targeted familial variant testing was used (20%, n=52/261). Other genetic tests included targeted testing for known Finnish founder variants (16%, n=43/261), Sanger sequencing of single or multiple genes (15%, n=38/261) and whole-exome sequencing (WES) (6%, n=16/261).

The identified variants were classified according to the American College of Medical Genetics and Association for Molecular Pathology variant interpretation guidelines.^5^ Pathogenic or likely pathogenic variants were considered clinically significant and calculated in the diagnostic yield analysis.

### Estimating the prevalence of IRD in Northern Finland

Prevalence per 100 000 inhabitants, with a 95% CI, was calculated for patients alive at the end of 2023. Population numbers for the target area were provided by Statistics Finland (www.stat.fi).

### Evaluating potential genetic therapeutic prospects

Information on current and future genetic therapy options was gathered from the European Medicines Agency (EMA, https://www.ema.europa.eu/en/), the Finnish Medicines Agency (Fimea, https://fimea.fi/) and ClinicalTrials.gov (https://clinicaltrials.gov/).

Details on syndromic IRDs, genes included in the NGS gene panels, less frequently used genetic tests, data analysis and gene therapy queries are available in the online supplemental methods.

## Results

### Clinical characteristics of the patients

The total study population was 58% male (n=338/582) and 42% female (n=244/582) (table 1). All patients were of Finnish ancestry. RP was the most prevalent phenotype (32%, n=185/582) (table 1). Gender distribution of different subphenotypes is presented in online supplemental table 1. Syndromic IRD was present in 19% of cases (n=112/582), with Usher syndrome and juvenile NCL being the most common syndromic forms (online supplemental table 2).

**Table 1:** Diagnostic yield stratified by gender and retinal subphenotype

	Out of total	Undergone genetic testing	Diagnostic yield
Male	58% (n=338/582)	56% (n=147/261)	83% (n=122/147)
Female	42% (n=244/582)	44% (n=114/261)	77% (n=88/114)
Phenotype			
Retinitis pigmentosa	32% (n=185/582)	39% (n=72/185)	68% (n=49/72)
X-linked retinoschisis	13% (n=78/582)	24% (n=19/78)	100% (n=19/19)
Usher syndrome	11% (n=65/582)	29% (n=19/65)	74% (n=14/19)
Choroideremia	9% (n=51/582)	43% (n=22/51)	91% (n=20/22)
Syndromic IRD*	8% (n=47/582)	66% (n=31/47)	100% (n=31/31)
Cone dystrophy/cone-rod dystrophy	8% (n=44/582)	55% (n=24/44)	63% (n=15/24)
Familial exudative vitreoretinopathy	6% (n=37/582)	100% (n=37/37)	86% (n=32/37)
Stargardt disease	4% (n=23/582)	39% (n=9/23)	66% (n=6/9)
Macular dystrophy	3% (n=16/582)	31% (n=5/16)	60% (n=3/5)
Severe early childhood-onset retinal dystrophy	2% (n=14/582)	93% (n=13/14)	100% (n=13/13)
Best disease	2% (n=10/582)	n<3	n<3
Achromatopsia	1% (n=4/582)	n<3	n<3
Congenital stationary night blindness	1% (n=3/582)	100% (n=3/3)	100% (n=3/3)
Wagner syndrome	1% (n=3/582)	0	0
Leber’s congenital amaurosis	n<3	n<3	n<3

*Syndromic IRD excluding Usher syndrome.

IRD, inherited retinal dystrophy.

### Genetic results

Pathogenic or likely pathogenic variants explaining IRD were identified in 36% (n=210/582) of all patients and 80% (n=210/261) of genetically tested patients with IRD. The pathogenic and likely pathogenic variants followed an autosomal recessive inheritance pattern in 45% (n=95/210) of cases (homozygous in 77%, n=73/95 and compound heterozygous in 23%, n=22/95), autosomal dominant in 20% (n=41/210), X-linked in 33% (n=70/210) and mitochondrial DNA-related inheritance in 2% (n=5/210).

**Figure 2:**
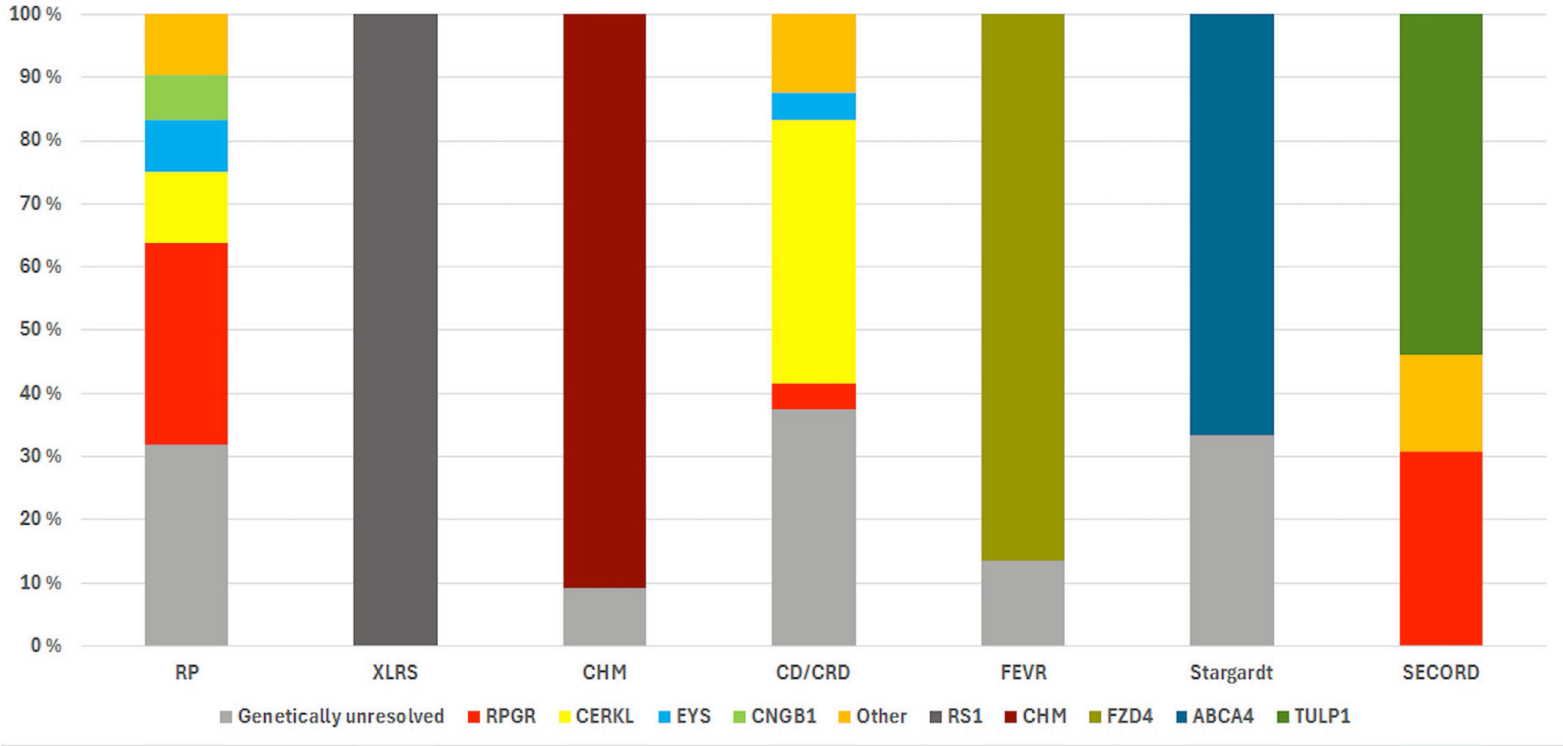
The distribution of genotypes and diagnostic yield in the seven most prevalent phenotypes of Northern Finnish patients with non-syndromic inherited retinal dystrophy. CD, cone dystrophy; CHM, choroideremia; CRD, cone-rod dystrophy; FEVR, familial exudative vitreoretinopathy; LCA, Leber’s congenital amaurosis; MD, macular dystrophy; RP, retinitis pigmentosa; SECORD, severe early childhood-onset retinal dystrophy; XLRS, X-linked retinoschisis.

**Figure 3:**
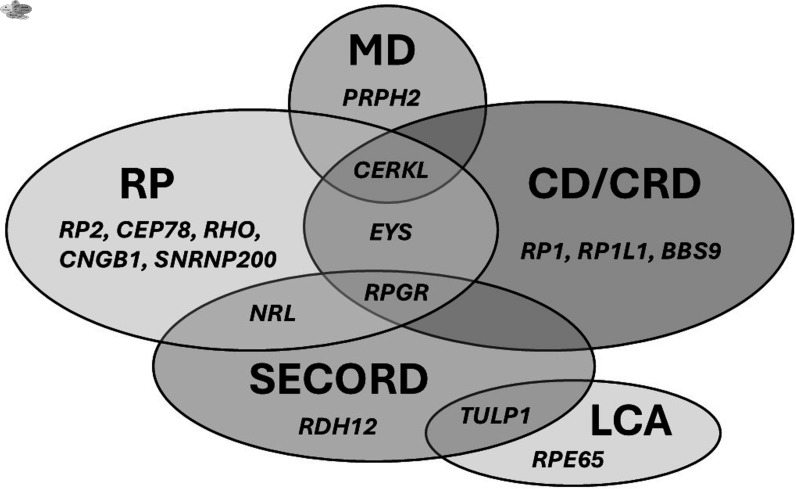
Clinical and genetic heterogeneity in Finnish patients with IRD. Phenotypes that did not overlap with multiple genes are not presented. CD, cone dystrophy; CRD, cone-rod dystrophy; LCA, Leber’s congenital amaurosis; MD, macular dystrophy; RP, retinitis pigmentosa; SECORD, severe early childhood-onset retinal dystrophy.

In total, 70 pathogenic or likely pathogenic variants were identified across 39 different genes, along with a large mitochondrial deletion (online supplemental table 3). The most common causative genes were *FZD4*, *RPGR*, *CHM*, *RS1*, *CERKL* and *CLRN1* (online supplemental figure 1). Additionally, 2% of all patients (n=9/582), 3% (n=9/261) of all genetically tested patients and 6% (n=7/124) of patients with IRD tested with NGS methods (gene panels or WES) had a variant of unknown significance (VUS) in an IRD-related gene. *RPGR* and *FZD4* genes were the most prevalent causes of IRD (online supplemental figure 2). RP, CD/CRD, MD, SECORD and LCA phenotypes demonstrated genetic heterogeneity (figure 2). Several genes, including *CERKL, EYS, RPGR, NRL* and *TULP1*, were associated with multiple subphenotypes (figure 3).

Among the 70 pathogenic or likely pathogenic variants, which manifested in 69 different combinations, 56% (n=39/70) were enriched in the Finnish population (online supplemental table 4). Five variants (7%, n=5/70) were classified as structural variants, defined as large genomic alterations encompassing at least 50 base pairs.^6^ Complex alleles in *EYS* and *ABCA4* were present in 4% (n=9/210) of patients. A nonsense mutation *EYS* c.1155T>A (p.Cys385*) co-occurred in cis with an 8-bp deletion (*EYS* c.8648_8655del, p.Thr2883Lysfs*4) and presented as a homozygote in 2% of patients (n=4/210), causing RP. *ABCA4* c.1622T>C (p.Leu541Pro) and c.3113C>T (p.Ala1038Val) presented in cis in 2% of patients (n=5/210) and formed a compound heterozygote with another pathogenic or likely pathogenic *ABCA4* variant, resulting in Stargardt disease.

### Diagnostic yield of genetic testing

The likelihood of identifying a pathogenic or likely pathogenic variant varied depending on the phenotype (table 1 and figure 2). In the phenotypic subanalysis, 39% (n=72/185) of patients with RP had undergone genetic testing, and 68% (n=49/72) of these genetically tested patients with RP had pathogenic or likely pathogenic variants in IRD-related genes. The genetic aetiology was most frequently confirmed in XLRS (100%, n=19/19), SECORD (100%, n=13/13), CSNB (100%, n=3/3) and CHM (91%, n=20/22). The lowest rates of identifying a pathogenic or likely pathogenic variant were observed for CD or CRD (63%, n=15/24) and MD (60%, n=3/5).

### Prevalence of inherited retinal disease in Northern Finland

As of 1 January 2024, there were 582 confirmed patients with IRD in Northern Finland. A total of 511 confirmed patients with IRD, ranging in age from 0 to 96 years, were alive and included in the prevalence estimation. Of the 732 255 people in the Northern region, 731 632 were aged 0–96 years. We estimated the prevalence of IRD in Northern Finland for individuals aged 0–96 years to be 1:1432 (69.8/100 000). The prevalence rates for each subphenotype are presented in table 2. For the X-linked recessive disorders, CHM and XLRS, prevalence was estimated for both genders. The most prevalent subphenotypes were RP, XLRS, Usher syndrome, CHM and CD/CRD, with prevalence rates of 25.3, 10.7, 8.9, 7 and 6 per 100 000, respectively. Among males, the prevalence of XLRS and CHM was 20.7 and 10.6 per 100 000, respectively.

**Table 2:** Estimated total prevalence of IRD and its subphenotypes in Northern Finland

	Prevalence (per 100 000)	CI
IRD	69.8	64.1 to 76.2
Retinitis pigmentosa	25.3	21.9 to 29.2
X-linked retinoschisis	10.7	8.5 to 13.3
Male	20.6	16.5 to 25.8
Female	0.55	0.15 to 2.0
Usher syndrome	8.9	7.0 to 11.3
Choroideremia	7.0	5.3 to 9.2
Male	10.6	7.7 to 16.9
Female	3.3	1.9 to 5.8
Cone dystrophy/cone-rod dystrophy	6.0	4.5 to 8.1
Syndromic IRD	5.5	4.0 to 7.4
Familial exudative vitreoretinopathy	5.1	3.7 to 7.0
Stargardt disease	3.1	2.1 to 4.7
Macular dystrophy	2.2	1.4 to 3.6
LCA and SECORD*	2.2	1.4 to 3.6
Best disease	1.4	0.74 to 2.5
Gyrata atrofia	0.96	0.46 to 2.0
Achromatopsia	0.55	0.21 to 1.4
Congenital stationary night blindness	0.41	0.14 to 1.2
Wagner syndrome	0.41	0.14 to 1.2

* The prevalence of LCA and SECORD is presented as a combined total.

IRD, inherited retinal dystrophy; LCA, Leber’s congenital amaurosis ; SECORD, severe early childhood-onset retinal dystrophy.

### Genes identified with potential therapeutic prospects

In this cohort, 18% of patients (n=38/210) had a pathogenic or likely pathogenic gene variant in a gene associated with either an ongoing clinical trial or an already EMA-approved gene therapy (online supplemental results).

## Discussion

We present a population-based registry of 582 clinically diagnosed patients with IRD from Northern Finland. In total, 70 pathogenic or likely pathogenic variants were identified across 39 genes, with several genes associated with multiple phenotypes, demonstrating the genetic and phenotypic heterogeneity of IRD. The overall diagnostic yield was 80% among genetically tested individuals, although this varied by subphenotype. The relatively high diagnostic yield in our study compared with previous studies may be due to the presence of known Finnish founder variants, which are highly prevalent in the population.^1 4 7^

Multiple IRD-associated variants were overrepresented in this cohort. This is likely due to the Finnish population history, characterised by multiple recent bottlenecks and genetic drift, followed by rapid population expansion. The most common causative gene was *FZD4*, associated with a Finnish founder variant.^8^ The most frequent cause of RP and the second most prevalent gene with pathogenic or likely pathogenic variants was *RPGR*. Globally, *RPGR* is a major IRD-associated gene, and a multicentre study of over 5000 patients with IRD from various countries identified *RPGR* as the third most common causative gene after *ABCA4* and *USH2A*.^9^

In this study, 7% (n=5/70) of the identified variants were structural or copy number variants. This finding aligns with previous research indicating that copy number variations contribute to 9% of pathogenic IRD cases.^10^ Notably, we identified an intragenic inversion in *CHM* that segregated with the choroideremia phenotype. Disruptive inversions in haploinsufficient genes are a known pathogenetic mechanism, highlighting the importance of systematically analysing these variants in standard diagnostic pipelines.^11^ To the best of our knowledge, pathogenic inversions of *CHM* have not been previously described as a cause for choroideremia, although an inverted duplication has been reported.^12^

Complex alleles in *ABCA4* and *EYS* were responsible for IRD in 4% of patients (n=9/210). The *ABCA4* complex allele c.1622T>C (p.Leu541Pro) and c.3113C>T (p.Ala1038Val) is a globally recognised cause of IRD.^13 14^ Similarly, the *EYS* complex allele c.1155T>A (p.Cys385*) and c.8648_8655del (p.Thr2883Lysfs*4) was previously identified in a Finnish study, where it was found in a homozygous state in three Finnish patients and suggested to represent a founder haplotype.^15^ *EYS* c.1155T>A (p.Cys385*), *EYS* c.8648_8655del (p.Thr2883Lysfs*4) and *ABCA4* c.1622T>C (p.Leu541Pro) variants were all enriched in the Finnish population.

In this study, 6% (n=7/124) of patients with IRD tested using NGS methods had a VUS in an IRD-related gene. Other studies have reported a higher percentage of VUS,^16 17^ which may be due to varying policies among genetic laboratories on reporting VUS.

The estimated total prevalence of IRDs in our study was 1 in 1432, significantly higher than the adjusted minimum prevalence of 1 in 3856 reported in a recent study from Southeast Norway.^6^ In 1994, the prevalence of XLRS in Finland was estimated to be 1 in 17 000.^18^ Our results estimate the prevalence of XLRS in Finland to be 1 in 9000, suggesting an increase possibly due to improved disease awareness among clinicians and advances in genetic testing. Our study estimates the prevalence of CHM in Finland to be 1 in 14 000, which is significantly higher than the estimated global prevalence of 1 in 50 000–100 000.^19^ Previous studies have indicated that Northern Finland has the highest prevalence of CHM, although exact figures have not been published.^20 21^

Interestingly, 18% of patients in this study had a pathogenic or likely pathogenic variant in a gene with either an ongoing clinical trial or an already EMA-approved gene therapy demonstrating the potential of gene therapy strategies in the treatment of IRDs. Among ongoing clinical trials for IRD, gene therapy targeting *RPGR* is the most studied. The future prospects of *RPGR*-targeted gene therapy are intriguing, considering the high global prevalence of *RPGR-*related IRD.

Over half of the study population was male (58%, n=338/582), largely due to the contribution of X-linked inherited IRD. We also identified symptomatic female patients with XLRS and CHM. While XLRS and CHM have been reported globally to occur almost exclusively in males, symptomatic female carriers have been documented, likely due to skewed X inactivation.^20 22 23^ Our results align with a recent study, showing that a proportion of female carriers of CHM has moderate to severe, progressive disease.^24^

This study has several limitations, primarily due to its retrospective nature. Patients underwent a variety of genetic testing approaches, and many had not undergone genetic testing at all. Another limitation is that we had to exclude 207 Finnish patients from the original study population of 805, possibly leading to an underestimated prevalence of IRD. However, prevalence estimations only include living patients, and nearly half (49%) of the excluded patients had died. Some excluded patients had an ICD-10 diagnosis suggestive of IRD, but a manual review of electronic patient records found insufficient clinical data to support the diagnosis. These patients may have been incorrectly assigned an IRD-related ICD-10 code, or they may have had a syndrome associated with incomplete IRD penetrance. In the future, a prospective study including comprehensive genetic testing and detailed ophthalmological phenotyping would provide more precise knowledge regarding the genetic aetiology of IRD in the Finnish population.

Achieving a genetic diagnosis of IRD has multiple advantages. It allows professionals to predict disease progression, enabling tailored follow-up care for patients. In patients with syndromic IRD, genetic information facilitates early detection of associated phenotypes, allowing for timely intervention and management. Genetic data also inform recurrence risk within families, and in cases of severe syndromic IRD, preimplantation testing or prenatal diagnostics may be considered upon parental request. The growing understanding of genetic aetiology and the development of gene therapies offer patients with IRD access to emerging treatment options.

In conclusion, this comprehensive study presents the unique genetic landscape and the first-ever estimated prevalence of IRD in the Northern Finnish population. While our results on genetic and phenotypic heterogeneity and differences in diagnostic yields of various subphenotypes can be generalised to other populations, the unique distribution of pathogenic variants is specific to the Finnish population. Approximately half of the disease-causing variants identified in this study were enriched in the Finnish population, underscoring the importance of conducting population-specific studies.

## Supplementary material

10.1136/bjo-2025-327427 online supplemental file 1

10.1136/bjo-2025-327427 online supplemental file 2

## Data Availability

All data relevant to the study are included in the article or uploaded as supplementary information.
