# Supplementary material for: Characterisation and prevalence of inherited retinal diseases in the Finnish population reveals enrichment of population-specific phenotypes and causative variants
Source: Br J Ophthalmol. 2025 Jun 26;109(8):e327427. doi: 10.1136/bjo-2025-327427 (PMC12320592; doi:10.1136/bjo-2025-327427)
Supplement: online supplemental file 1 [file bjo-109-8-s001.docx]

**SUPPLEMENTAL MATERIAL**

**Characterisation and prevalence of inherited retinal diseases in the Finnish population reveals enrichment of population-specific phenotypes and causative variants**

Laura Lähteenoja^1,2,3^, Pasi Ohtonen^4^, Aura Falck^1,3,^ , Elisa Johanna Rahikkala^1,2,5^

^1^ Research Unit of Clinical Medicine and Medical Research Center Oulu, Oulu University Hospital and University of Oulu, Oulu, Finland

^2^ Clinical Genetics Unit, Oulu University Hospital, Oulu, Finland

^3^ Ophthalmology Unit, Oulu University Hospital, Oulu, Finland

^4^ Research Service Unit, Oulu University Hospital and Translational Medicine Research Unit, University of Oulu, Oulu, Finland

^5^ Genomics Unit, Turku University Hospital, Turku, Finland

Elisa Rahikkala and Aura Falck contributed equally as senior authors

**SUPPLEMENTAL METHODS**

*Clinical evaluation*

Syndromic IRDs included patients with gyrate atrophy, Cockayne syndrome, Bardet–Biedl syndrome, infantile neuronal ceroid lipofuscinosis (INCL), neuronal ceroid lipofuscinosis type 5 (NCL5), juvenile neuronal ceroid lipofuscinoses (JNCL), MELAS syndrome, other mitochondrial diseases, Coats plus syndrome, Aicardi syndrome, Cohen syndrome, neuromuscular oculoauditory syndrome (NMOAS), neurodevelopmental disorder with retinitis pigmentosa, and long-chain 3-hydroxyacyl-CoA dehydrogenase (LCHAD) deficiency. Usher syndrome was reviewed as its own category due to its high prevalence in the study population compared to other syndromic IRDs.

Most IRD patients in this study were clinically examined by retina specialists at Oulu University Hospital, a tertiary referral centre for Northern Finland. Some patients were assessed by ophthalmologists at a central hospital. Over the decades covered by this study, dilated fundus examinations, fundus photography, dark adaptation tests, and visual field testing were routinely performed. Since the late 1990s, electroretinograms (ERG) and, later, optical coherence tomography (OCT) have been included in the routine diagnostic work-up. Patients whose IRD diagnoses were not clearly documented in their medical records were excluded from the study.

*Genetic tests used*

Less frequently used genetic tests included whole-genome sequencing (WGS) (n<3), targeted exon array comparative genomic hybridisation of the *CHM* gene (n<3), extra long polymerase chain reaction (XL-PCR) of the mitochondrial DNA method (n<3), and microchip techniques (n<3). ‘n<3’ indicates that the test was conducted on one or two individuals. Due to the nature of this study, individualised data cannot be presented.

*NGS gene panel content*

The gene panels performed vary depending on the laboratory where the test is conducted and the year the test is ordered as the NGS gene panels are regularly updated. Gene panel testing started in 2013 and during 2013–2018 IRD patient samples were sent to Manchester Centre for Genomic Medicine laboratory, U.K. This gene panel included during these years 105–176 genes depending on the panel version used. Later on, samples were sent to Blueprint Genetics laboratory, Espoo, Finland. The Blueprint Genetics Retinal Dystrophy Panel gene panel used during these years included 181–351 genes.

The list of genes included in the Manchester Centre for Genomic Medicine laboratory, U.K. NGS gene panel in 2018: ABCA4; ABHD12; ACBD5; ADAM9; ADAMTS18; AHI1; AIPL1; ARL2BP; ARL6; BBIP1; BBS1; BBS10; BBS12; BBS2; BBS4; BBS5; BBS7; BBS9; BEST1; C1QTNF5; C2orf71; C2ORF86/(WDPCP); C8ORF37; C21orf2; CA4; CABP4; CACNA1F; CACNA2D4; CAPN5; CC2D2A; CDH3; CDH23; CDHR1; CEP164; CEP290; CERKL; CHM; CIB2; CLN3; CLRN1; CNGA1; CNGA3; CNGB1; CNGB3; CNNM4; CRB1; CRX; CSPP1; CYP4V2; DFNB31; DHDDS; DTHD1; EFEMP1; ELOVL4; EMC1; EYS; FAM161A; FLVCR1; FSCN2; FZD4; GNAT1; GNAT2; GNPTG; GPR125; GPR179; GPR98; GRM6; GUCA1A; GUCA1B; GUCY2D; HARS; HMX1; IDH3B; IFT140; IMPDH1; IMPG1; IMPG2 7; INPP5E; INVS; IQCB1; ITM2B; KCNJ13; KCNV2; KIAA1549; KIF11; KLHL7; LCA5; LRAT; LRP5; LZTFL1; MERTK; MFRP; MKKS; MKS1; MVK; MYO7A; NDP; NEK2; NMNAT1; NPHP1; NPHP3; NPHP4; NR2E3; NRL; NYX; OAT; OFD1; OTX2; PANK2; PCDH15; PCYT1A; PDE6A; PDE6B; PDE6C; PDE6G; PEX1; PEX2/(PXMP3); PEX7; PHYH; PITPNM3; PLA2G5; PRCD; PROM1; PRPF3; PRPF31; PRPF4; PRPF6; PRPF8; PRPH2; RAB28; RAX2; RBP3; RBP4; RD3; RDH12; RDH5; RGR; RGS9; RHO; RIMS1; RLBP1; ROM1; RP1; RP1L1 (excluding exon 4); RP2; RP9; RPE65; RPGR (excluding ORF15); RPGRIP1; RPGRIP1L; RS1; SAG; SDCCAG8; SEMA4A; SLC24A1; SNRNP200; SPATA7; TEAD1; TIMP3; TMEM237; TOPORS; TRIM32; TRPM1; TSPAN12; TTC8; TUB; TULP1; UNC119; USH1C; USH1G; USH2A; VCAN; VPS13B; WDR19; ZNF423; ZNF513

The latest version of the Blueprint Genetics Retinal Dystrophy Panel (version 7, Oct 30, 2021) Plus Analysis includes sequence analysis and copy number variation analysis of the following genes: ABCA4, ABCC6, ABCD1, ABHD12, ACO2, ADAM9, ADAMTS18, ADGRV1, ADIPOR1, AGBL5, AHI1, AIPL1, ALMS1, AMACR, ARHGEF18, ARL13B, ARL2BP, ARL3, ARL6, ARMC9, ARR3, ARSG, ATF6, ATOH7, B9D1, B9D2, BBIP1, BBS1, BBS10, BBS12, BBS2, BBS4, BBS5, BBS7, BBS9, BEST1, C1QTNF5, C21ORF2, C2ORF71, C5ORF42, C8ORF37, CA4, CABP4, CACNA1F, CACNA2D4, CAPN5, CC2D2A, CDH23, CDH3, CDHR1, CEP104, CEP120, CEP164, CEP19, CEP250, CEP290, CEP41, CEP78, CEP83, CERKL, CHM, CIB2, CISD2, CLN3, CLN5, CLN6, CLN8, CLRN1, CNGA1, CNGA3, CNGB1, CNGB3, CNNM4, COL11A1, COL11A2, COL18A1, COL2A1, COL9A1, COL9A2, COL9A3, COQ2, CPE, CRB1, CRX, CSPP1, CTC1, CTNNA1, CTNNB1, CTSD, CWC27, CYP4V2, DFNB31, DHDDS, DHX38, DNAJC5, DRAM2, DTHD1, DYNC2H1, EFEMP1, ELOVL4, EMC1, ESPN, EXOSC2, EYS, FAM161A, FDXR, FLVCR1, FRMD7, FZD4, GNAT1, GNAT2, GNB3, GNPTG, GPR143, GPR179, GRK1, GRM6, GUCA1A, GUCY2D, HARS, HGSNAT, HK1, HMX1, IDH3A, IDH3B, IFT140, IFT172, IFT27, IFT81, IMPDH1, IMPG1, IMPG2, INPP5E, INVS, IQCB1, ISPD, JAG1, KCNJ13, KCNV2, KIAA0556, KIAA0586, KIAA0753, KIAA1549, KIF11, KIF7, KIZ, KLHL7, LAMA1, LCA5, LRAT, LRIT3, LRP2, LRP5, LZTFL1, MAK, MERTK, MFN2, MFRP, MFSD8, MKKS, MKS1, MMACHC, MT-ATP6, MT-ATP8, MT-CO1, MT-CO2, MT-CO3, MT-CYB, MT-ND1, MT-ND2, MT-ND3, MT-ND4, MT-ND4L, MT-ND5, MT-ND6, MT-RNR1, MT-RNR2, MT-TA, MT-TC, MT-TD, MT-TE, MT-TF, MT-TG, MT-TH, MT-TI, MT-TK, MT-TL1, MT-TL2, MT-TM, MT-TN, MT-TP, MT-TQ, MT-TR, MT-TS1, MT-TS2, MT-TT, MT-TV, MT-TW, MT-TY, MTTP, MVK, MYO7A, NAGLU, NDP, NEK2, NMNAT1, NPHP1, NPHP3, NPHP4, NR2E3, NR2F1, NRL, NYX, OAT, OCA2, OFD1, OPA1, OPA3, OPN1SW, OTX2, P3H2, PANK2, PAX2, PCDH15, PCYT1A, PDE6A, PDE6B, PDE6C, PDE6D, PDE6G, PDE6H, PDSS1, PDSS2, PDZD7, PEX1, PEX10, PEX11B, PEX12, PEX13, PEX14, PEX16, PEX19, PEX2, PEX26, PEX3, PEX5, PEX6, PEX7, PHYH, PISD, PITPNM3, PLA2G5, PLK4, PNPLA6, POC1B, POMGNT1, PPT1, PRCD, PRDM13, PROM1, PRPF3, PRPF31, PRPF4, PRPF6, PRPF8, PRPH2, PRPS1, RAB28, RAX2, RBP3, RBP4, RCBTB1, RD3, RDH11, RDH12, RDH5, REEP6, RGR, RGS9, RGS9BP, RHO, RIMS1, RLBP1, ROM1, RP1, RP1L1, RP2, RPE65, RPGR, RPGRIP1, RPGRIP1L, RS1, RTN4IP1, SAG, SAMD11, SCAPER, SCLT1, SDCCAG8, SEMA4A, SGSH, SLC24A1, SLC25A46, SLC45A2, SLC7A14, SNRNP200, SPATA7, SPP2, SRD5A3, TCTN1, TCTN2, TCTN3, TEAD1, TIMM8A, TIMP3, TMEM107, TMEM126A, TMEM138, TMEM216, TMEM231, TMEM237, TMEM67, TOPORS, TPP1, TRAF3IP1, TREX1, TRIM32, TRPM1, TSPAN12, TTC21B, TTC8, TTLL5, TTPA, TUB, TUBB4B, TUBGCP4, TUBGCP6, TULP1, TYR, TYRP1, USH1C, USH1G, USH2A, VCAN, VPS13B, WDPCP, WDR19, WFS1, YME1L1, ZNF408, ZNF423 and ZNF513.

*Data analysis*

The identified variants were classified according to the American College of Medical Genetics and Association for Molecular Pathology (ACMG/AMP) variant interpretation guidelines.^1^ Population frequencies of the variants were obtained from the Genome Aggregation Database (GnomAD v.4.1.0).^2^ Variants were considered Finnish-enriched if their allele frequency in the GnomAD v4 Finnish population was at least threefold higher than in any other population or if they were absent from GnomAD v4 (i.e., present only in the Finnish population).

*Evaluating potential genetic therapeutic prospects*

Present genetic therapy options for Finnish patients with inherited retinal dystrophies (IRDs) were reviewed through European Medicines Agency (EMA, https://www.ema.europa.eu/en/) and the Finnish Medicines Agency (Fimea, https://fimea.fi). To identify potential therapeutic prospects, a query was conducted on ClinicalTrials.gov (https://clinicaltrials.gov/) on November 25, 2024, to determine which of the genes identified in this study had active gene therapy clinical trials. The search included the disease term ‘inherited retinal dystrophy’ (as provided by the website) and ‘gene therapy’ as the intervention/treatment.

**SUPPLEMENTAL RESULTS**

*Genes identified with potential therapeutic prospects*

Currently, an EMA-approved drug, voretigene neparvovec, is available for genetically confirmed RPE65 deficiency (www.ema.europa.eu), In our study population, pathogenic variants in *RPE65* were a rare cause of IRD (n<3). A search of ongoing clinical trials on ClinicalTrials.gov identified 58 relevant studies. Of these, 13 were clinical trials applying gene therapy as an intervention for IRD caused by pathogenic variants in *RPGR*. Six trials involved *RPE65*, two targeted *RHO*, one focused on *USH2A*, and one addressed *ABCA4*. The remaining clinical trials targeted genes that were either not relevant to our study population or did not specify which gene the genetic therapy targeted.

**REFERENCES**

1 Richards S, Aziz N, Bale S, *et al.* Standards and guidelines for the interpretation of sequence variants: a joint consensus recommendation of the American College of Medical Genetics and Genomics and the Association for Molecular Pathology. *Genet Med Off J Am Coll Med Genet*. 2015;17:405–24. doi: 10.1038/gim.2015.30

2 Chen S, Francioli LC, Goodrich JK, *et al.* A genomic mutational constraint map using variation in 76,156 human genomes. *Nature*. 2024;625:92–100. doi: 10.1038/s41586-023-06045-0

**Supplemental Figures**

**Supplemental Figure 1** The most common causative genes. Genes causing IRD for one or two patients were *RP2*, *CEP78*, *NRL*, *CACNA1F*, *RPE65*, *RP1*, *RP1L1*, *PRPH2*, *SNRNP200*, *HK1*, *BEST1*, *GRM6*, *BBS9*, *CNGB3*, *PDE6C*, *BBS1*, *BBS2*, *ERCC6*, *PPT1*, *USH2A*, *RHO*, *DHX16*, and *RDH12*.


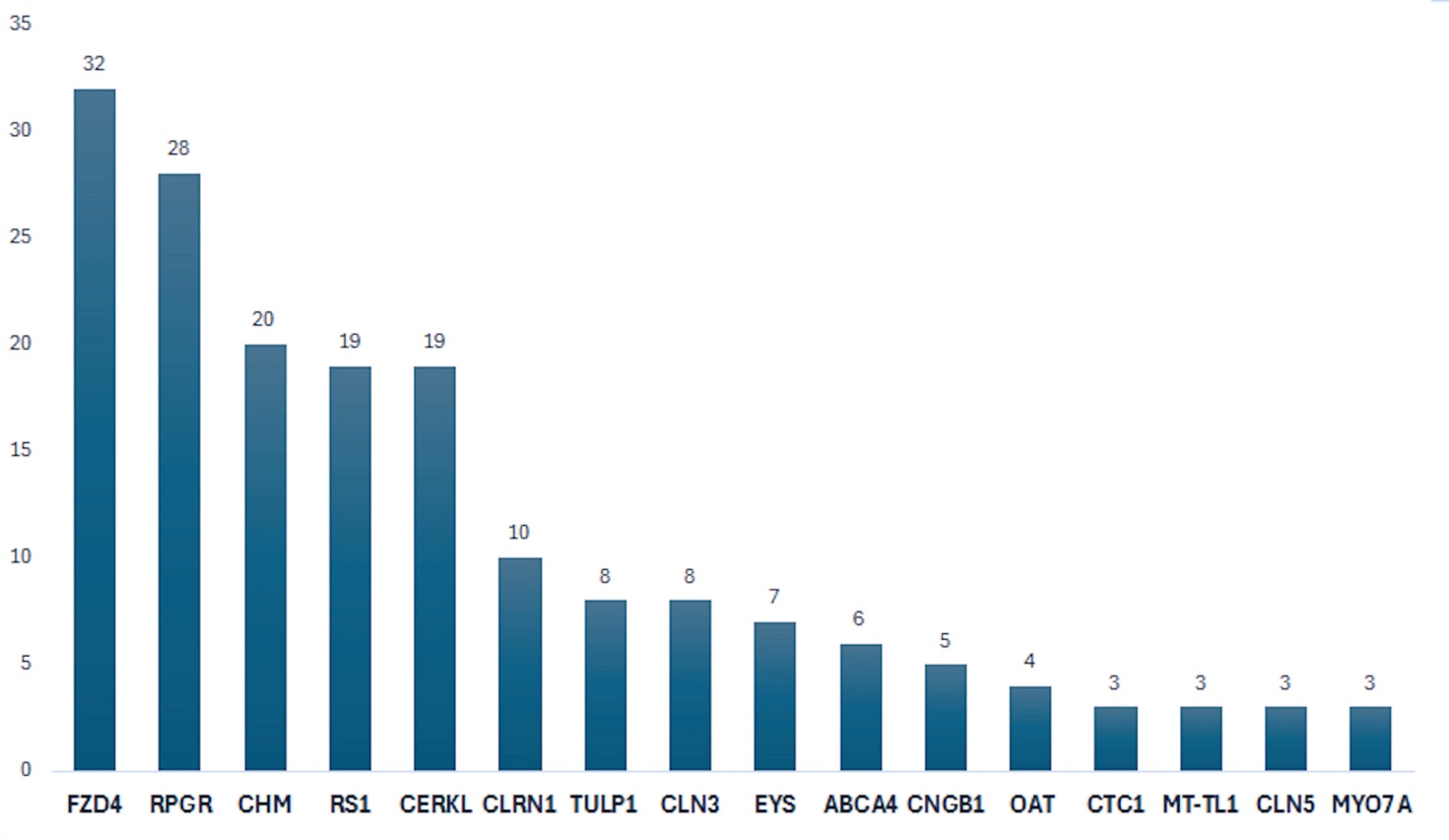


**Supplemental Figure 2** The most prevalent genes with likely pathogenic variants in patients with non-syndromic IRD (n=166). ‘Other’ refers to genes with pathogenic variants identified in one or two patients (n=22).


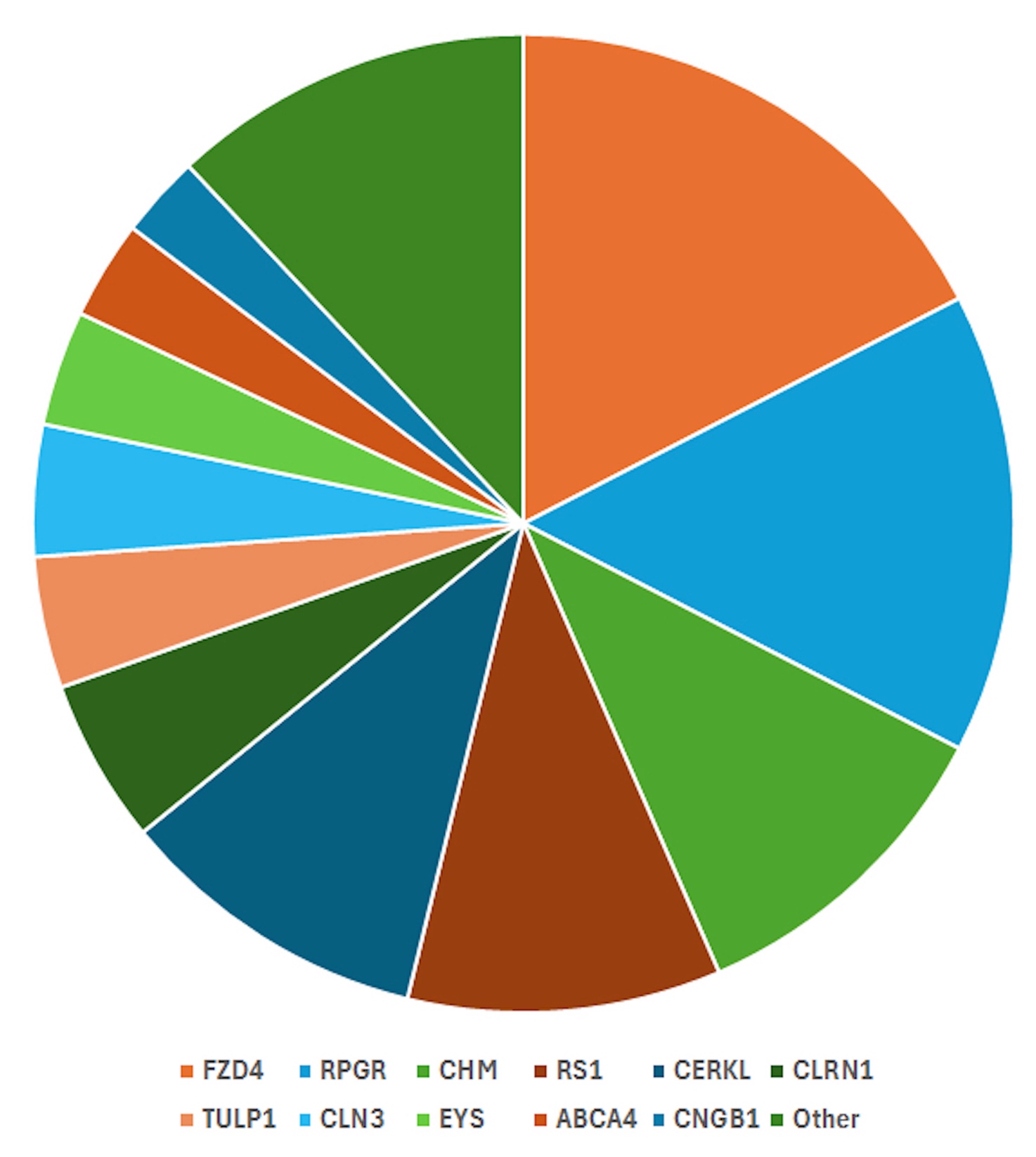


**Supplemental Tables**

| **Supplemental Table 1.** Gender distribution in non-syndromic IRD phenotypes | | | |
| --- | --- | --- | --- |
| *Phenotype* | *Number of affected* | *Male* | *Female* |
| RP | 185 | 51% (n=94/185) | 49% (n=91/185) |
| XLRS | 78 | 97% (n=76/78) | 3% (n=2/78) |
| Usher syndrome | 65 | 42% (n=27/65) | 58% (n=38/65) |
| CHM | 51 | 76% (n=39/51) | 24% (n=12/51) |
| CD/CRD | 44 | 61% (n=27/44) | 39% (n=17/44) |
| FEVR | 37 | 46% (n=17/37) | 54% (n=20/37) |
| Stargardt disease | 23 | 48% (n=11/23) | 52% (n=12/23) |
| MD | 16 | 44% (n=7/16) | 56% (n=9/16) |
| SECORD | 14 | 36% (n=5/14) | 64% (n=9/14) |
| Best disease | 10 | 50% (n=5/10) | 50% (n=5/10) |
| Other | 12 |  |  |
| Phenotypes with <3 males were LCA, CSNB, achromatopsia, and Wagner syndrome. | | | |

| **Supplemental Table 2.** Number of patients with syndromic IRD subphenotypes | |
| --- | --- |
| *Syndromic IRD phenotype* | *Number of affected patients*  *(total n=112)* |
| Usher syndrome | 65 |
| JNCL | 8 |
| INCL | 7 |
| Gyrate atrophy | 7 |
| Cohen syndrome | 5 |
| Coats plus syndrome | 4 |
| NCL5 | 3 |
| MELAS syndrome | 3 |
| Bardet-Biedl syndrome  Cockayne syndrome, NMOAS, Aicardi syndrome, LCHAD deficiency, mitochondrional disease, neurodevelopmental disorder with retinitis pigmentosa | 3  n<3 |
| n<3 means 1 or 2 individuals. Due to the nature of this study, individualized level of data cannot be presented. | |

| **Supplemental Table 4.** Finnish-enriched variants | | | | | |
| --- | --- | --- | --- | --- | --- |
| ***Gene*** | ***Variant*** | ***Transcript*** | ***GnomAD v4.1.0 freq in Finns*** | ***GnomAD freq in non-Finnish Europeans*** | ***Allele freq in Finns / Allele freq in non-Finnish Europeans*** |
| **AR-inherited** | | | | | |
| *NRL* | c.529C>A | NM_001354768.3 | 0.00001874 | 0.000 |  |
| *RP1* | c.121T>C | NM_006269.2 | 0.0004998 | 0.000005932 | 84x |
| *RP1* | c.515T>G | NM_006269.2 | 0.0004377 | 0.00005508 | 7x |
| *EYS* | c.1155T>A | NM_001142800.2 | 0.005962 | 0.00004984 | 119x |
| *EYS* | c.8648_8655del | NM_001142800.2 | 0.006617 | 0.000129 | 51x |
| *ABCA4* | c.1622T>C | NM_000350.3 | 0.0006091 | 0.000139 | 4x |
| *ABCA4* | c.1610G>A | NM_000350.3 | 0.01262 | 0.0008542 | 14x |
| *TULP1* | c.148del | NM_003322.6 | 0.00255 | 0.000001888 | 1350x |
| *CERKL* | c.193G>T | NM_201548.5 | 0.001277 | 0.000007702 | 165x |
| *CERKL* | c.375C>G | NM_201548.5 | 0.008344 | 0.00002459 | 339x |
| *CNGB1* | c.2957A>T | NM_001297.5 | 0.006294 | 0.001141 | 5x |
| *GRM6* | c.712C>T | NM_000843.4 | 0.001049 | 0.00005680 | 18x |
| *BBS9* | c.728A>G | NM_198428.3 | 0.001297 | 0.00001526 | 84x |
| *PDE6C* | c.1670G>A | NM_006204.4 | 0.0008434 | 0.000001695 | 497x |
| *BBS1* | c.724-1G>C | NM_024649.5 | 0.0009370 | 0.000005086 | 184x |
| *CTC1* | c.680C>T | NM_025099.6 | 0.001235 | 0.0000008475 | 1457x |
| *CTC1* | c.2831del | NM_025099.6 | 0.003311 | 0.00008051 | 41x |
| *CTC1* | c.1994T>G | NM_025099.6 | 0.002343 | 0.000 |  |
| *CTC1* | c.3425_3426delinsAT | NM_025099.6 | 0.0008901 | 0.000004237 | 210x |
| *ERCC6* | c.3862C>T | NM_000124.4 | 0.001218 | 0.00005254 | 23x |
| *OAT* | c.1205T>C | NM_000274.4 | 0.002562 | 0.000009322 | 274x |
| *OAT* | c.539G>C | NM_000274.4 | 0.0005935 | 0.00002712 | 21x |
| *CLN3* | c.1001G>A | NM_001042432.2 | 0.0001720 | 0.00002034 | 8x |
| *PPT1* | c.364A>T | NM_000310.4 | 0.006907 | 0.00004494 | 153x |
| *MYO7A* | c.1623dupC | NM_000260.4 | 0.00005002 | 0.000 |  |
| *MYO7A* | c.2766_2779del | NM_000260.4 | 0.0009855 | 0.000005942 | 165x |
| *USH2A* | c.3367T>C | NM_206933.4 | 0.0005001 | 0.000001695 | 295x |
| *CLRN1* | c.528T>G | NM_174878.3 | 0.005950 | 0.00005170 | 115x |
| **XL-inherited** | | | | | |
| *RPGR* | c.778+5G>A | NM_001034853.2 | 0.00002151 | 0.000 |  |
| *RPGR* | c.679C>T | NM_001034853.2 | 0.00002138 | 0.000 |  |
| *CHM* | c.1609+2dup | NM_000390.4 | 0.00004300 | 0.000 |  |
| *CHM* | c.1006_1007del | NM_000390.4 | 0.00002134 | 0.000 |  |
| *RS1* | c.325G>C | NM_000330.4 | 0.0001921 | 0.000001117 | 171x |
| *RS1* | c.214G>A | NM_000330.4 | 0.00004269 | 0.000002234 | 19x |
| **AD-inherited** | | | | | |
| *FZD4* | c.313A>G | NM_012193.4 | 0.0001252 | 0.00001356 | 9x |
| *RP1L1* | c.3599G>A | NM_178857.6 | 0.00003141 | 0.000 |  |
| *PRPH2* | c.136C>T | NM_000322.5 | 0.00003124 | 0.00000339 | 9x |
| *SNRNP200* | c.2041C>T | NM_014014.5 | 0.00001562 | 0.000 |  |
| *BEST1* | c.85T>C | NM_004183.4 | 0.00001574 | 0.000 |  |
| Only present in Finns; freq., frequency | | | | | |
